# In Situ Formed Ag‐Li Intermetallic Layer for Stable Cycling of All‐Solid‐State Lithium Batteries

**DOI:** 10.1002/advs.202103826

**Published:** 2021-11-21

**Authors:** Hong Jun Choi, Dong Woo Kang, Jun‐Woo Park, Jun‐Ho Park, Yoo‐Jin Lee, Yoon‐Cheol Ha, Sang‐Min Lee, Seog Young Yoon, Byung Gon Kim

**Affiliations:** ^1^ Next Generation Battery Research Center Korea Electrotechnology Research Institute (KERI) 12, Jeongiui‐gil, Seongsan‐gu Changwon‐si Gyeongsangnam‐do 51543 Republic of Korea; ^2^ School of Materials Science and Engineering Pusan National University 2, Busandaehak‐ro 63beon‐gil, Geumjeong‐gu Busan 46241 Republic of Korea; ^3^ Graduate Institute of Ferrous and Energy Materials Technology Pohang University of Science and Technology (POSTECH) 77 Cheongam‐Ro, Nam‐gu Pohang Gyeongbuk 37673 Republic of Korea

**Keywords:** Ag‐Li, all‐solid‐state‐batteries, dendrite‐free, intermetallic layer, roll pressing

## Abstract

With the timely advent of the electric vehicle era, where battery stability has emerged as a major issue, all‐solid‐state batteries (ASSBs) have attracted significant attention as the game changer owing to their high stability. However, despite the introduction of a densely packed solid electrolyte (SE) layer, when Li is used to increase the energy density of the cell, the short‐circuit problem caused by Li protrusion is unavoidable. Furthermore, most strategies to control nonuniform Li growth are so complicated that they hinder the practical application of ASSBs. To overcome these limitations, this study proposes an Ag‐Li alloy anode via mass‐producible roll pressing method. Unlike previous studies reporting solid‐solution‐based metal alloys containing a small amount of lithiophilic Ag, the in situ formed and Ag‐enriched Ag‐Li intermetallic layer mitigates uneven Li deposition and maintains a stable SE/Ag‐Li interface, facilitating reversible Li operation. Contrary to Li cells showing frequent initial short‐circuit, the cell incorporating the Ag‐Li anode exhibits a better capacity retention of 94.3% for 140 cycles, as well as stable cycling even under 12 C. Through a facile approach enabling the fabrication of a large‐area anode with controllable Li growth, this study provides practical insight for developing ASSBs with stable cyclabilities.

## Graphical abstract

Compared to the sulfide‐based all‐solid‐state battery with Li metal anode exhibiting frequent initial short‐circuits, the cell incorporating Ag‐Li alloy anode fabricated via mass‐producible roll pressing method shows better cycle and rate performances, owing to the in situ formed Ag‐Li intermetallic layer physically controlling uneven Li deposition and chemically forming a stable solid electrolyte/Ag‐Li interface.

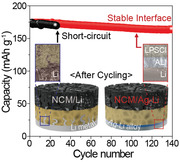

## 1 Introduction

**Scheme 1:**
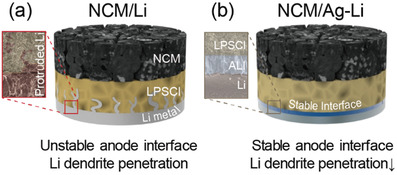
Comparative schematic illustration of ASSBs with a) Li metal and b) Ag‐Li alloy after cycling.

With the increasing demand for higher energy storage and safety systems, sulfide‐based all‐solid‐state batteries (ASSBs) comprising solidified and nonflammable components have been attracting strong interest owing to their potential in permitting the use of metallic Li because Li metal anodes would significantly increase the energy densities of ASSBs compared to their intercalation‐based counterparts using liquid electrolytes.^[^ ^1^, ^2^, ^3^, ^4^, ^5^, ^6^ ^]^ This application of ASSBs is because of the idea that solid electrolytes (SEs) act as a strong barrier that can physically prevent Li protrusion inside the SE, owing to their high shear modulus.^[^ ^7^, ^8^ ^]^ However, despite their excellent physical properties, short‐circuit caused by the uncontrollable Li growth in ASSBs has not been perfectly prevented thus far (**Scheme** **1**a), because metallic Li seeds first tend to be formed at inhomogeneous Li/SSE interfaces with interfacial voids and subsequent Li favors to be deposited at these seeds during successive charging processes, eventually resulting in rapid Li growth through the SE.^[^ ^7^, ^9^, ^10^, ^11^, ^12^ ^]^ Moreover, some studies have reported that, once a crack is generated by stress due to the inevitable volume change of the cell during cycling, and propagated inside the SEs by the Li filament growth, a short‐circuit would occur.^[^ ^12^, ^13^, ^14^, ^15^, ^16^ ^]^ Therefore, to circumvent this chronic issue, various approaches for introducing Li host and defect‐free densely packed SEs, and generating protective layers including artificial solid state interphase have been attempted.^[^ ^4^, ^5^, ^6^, ^17^, ^18^ ^]^ Nevertheless, these approaches have limitations in reducing dead volume in the 3D host, forming dense SE layers without any physical defects, and making the fabrication process easier. Consequently, most studies have continued to use Li‐In alloy as an anode material for ASSBs, because it shows dendrite‐free feature and higher interfacial stability compared to the metallic Li.^[^ ^19^, ^20^, ^21^ ^]^ However, despite these advantages, when assuming that the specific capacities of In and Li cells are the same, because In shows a higher operating voltage (≈0.6 V vs Li/Li^+^) compared to Li,^[^ ^20^ ^]^ it renders a difficult implementation of high‐energy‐density ASSBs. Accordingly, it is desirable to develop an anode with a low operating potential, dendrite‐free feature, and an easy fabrication process.

In this regard, to control the growth of Li dendrites in liquid‐based Li‐metal batteries, the concept of lithiophilicity has been widely introduced,^[^ ^22^, ^23^, ^24^, ^25^, ^26^, ^27^, ^28^ ^]^ which demonstrated a reduced nucleation barrier and suppressed Li dendrite growth by forming a solid solution buffer layer. In addition, Li‐containing structures with lithiophilic materials, such as Au, Ag, and Zn, showed improved electrochemical performance by controlling the direction of Li growth based on the strong lithiophilicity.^[^ ^22^, ^23^, ^24^ ^]^ In the same context, recently, anode‐free ASSBs incorporating pinhole‐free SE and lithiophilic Ag exhibited reversible Li plating/stripping processes and outstanding cycling performance, based on the solid solution process.^[^ ^26^, ^28^ ^]^ However, to achieve this, additional processes, including ultrahigh pressing processes such as warm isostatic pressing and additional electrode manufacturing processes, need to be applied.

With these concerns and investigations in mind, in the present investigation, we introduced an Ag‐Li alloy anode containing an in situ formed Ag‐Li intermetallic layer (ALI) via a mass‐producible roll pressing process using Ag and Li foils, to form a stable interface at the anode side (Scheme 1b). Contrary to the solid solution reaction wherein a small amount of Ag is dissolved in Li, the ALI contains a considerable amount of Ag, which enabled the formation of a stable interface with sulfide SEs and suppressed Li dendrite growth. Additionally, the residual small amount of Ag that does not form an ALI participated in the solid solution reaction with Li, thus alleviating the uneven Li growth. Owing to these attractive features, the cell incorporating ALI showed an improved capacity retention of 94.3% for 140 cycles, as well as stable cycling even under the harsh condition of 12 C, compared to the cell with metallic Li frequently suffering from an initial short‐circuit. This approach indicates that introducing a functional layer that can be in situ formed is an easy and effective way to improve the performance of ASSBs by maintaining stable anode interface and preventing Li protrusion during cycling.

## 2 Results and Discussion

**Figure 1:**
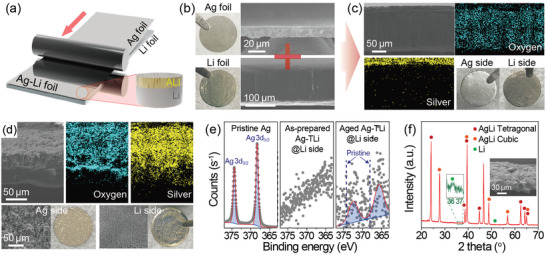
a) Experimental scheme for preparing Ag‐Li alloy foil. b) Cross‐sectional SEM images of the (upper) pristine Ag and (bottom) thick Li foils with corresponding digital photographs. Cross‐sectional SEM images and digital photographs of the c) as‐prepared Ag‐TLi and d) aged Ag‐TLi, and their corresponding EDS elemental mappings of oxygen and silver. Top‐view SEM images of Ag and Li sides of the aged Ag‐TLi are also displayed in (d). e) Ag 3d XPS spectra of the pristine Ag and Li sides of the as‐prepared and aged Ag‐TLi. f) XRD pattern of the aged Ag‐tLi shown in SEM image (inset).

Unlike previous complicated electrochemical or melting processes, to prepare the Ag‐Li alloy anode, 10 and 150 µm of Ag and Li (thick Li, TLi), respectively, were combined (denoted as Ag‐TLi) using a simple roll pressing method (**Figure** **1**a,b). The morphology of the as‐prepared Ag‐Li was visualized through scanning electron microscopy (SEM) analysis; it was confirmed that Ag and Li were only physically bound to each other (Figure 1c). However, after aging for 2 days under external pressure, the morphology of Ag‐Li changed due to the alloying reaction originating from the large difference in the Fermi energy level between Ag and Li (Ag (4.26 eV) vs Li (2.9 eV)).^[^ ^29^ ^]^ A digital image of the Ag side of the Ag‐Li alloy in Figure 1d shows the color change from silver to beige, and the cross‐sectional and top‐view SEM images of the Ag‐Li displayed newly and in situ formed ALI. In addition, contrary to the as‐prepared Ag‐Li, energy‐dispersive X‐ray spectroscopy (EDS) elemental mapping exhibited that a lithiophilic Ag element was found at the Li side below the ALI. To identify whether a small amount of Ag alloyed with Li, Ag 3d X‐ray photoelectron spectroscopy (XPS) analysis was conducted on the Li side of the aged Ag‐Li sample. As shown in Figure 1e, although Ag was not detected in the as‐prepared case, the aged Ag‐Li alloy exhibited broadened and shifted peaks at 365.8 and 372.3 eV associated with Ag 3d_5/2_ and Ag 3d_3/2_, respectively, compared to the pristine Ag metal;^[^ ^25^, ^29^ ^]^ this coherently indicates the existence of Ag in the Li metal and formation of Ag‐Li alloy. Furthermore, the top‐view EDS mapping images of the as‐prepared sample (Figure S1, Supporting Information), showing a small amount of Ag on the Li side, were well consistent with these spectroscopic data. To identify the phase of the in situ formed ALI, X‐ray diffraction (XRD) analysis was performed, and a 40 µm thick Li foil (thin Li, tLi) was used to make an ALI with minimized residual Li (denoted as Ag‐tLi), so as to mainly focus on the ALI. According to the XRD spectrum in Figure 1f, apart from a residual small Li peak at 36.19°, the peaks assigned to the AgLi intermetallic compound composed of almost the same molar ratio as Ag and Li were observed, because Ag_0.5_Li_0.5_ thermodynamically shows the lowest formation enthalpy.^[^ ^30^, ^31^ ^]^ Moreover, it was confirmed that the tetragonal and cubic phases existed together, although the tetragonal phase is known to be formed more favorably than the cubic phase,^[^ ^30^, ^32^ ^]^ which is in contrast to the composition with a small amount of Ag, exhibiting a solid solution behavior.^[^ ^26^, ^28^ ^]^ Besides, although pure Ag has a high electrical conductivity, its value is largely reduced by the structural and composition changes when forming an intermetallic compound,^[^ ^33^ ^]^ which would be indeed beneficial for the alleviation of Li plating on the top surface of the ALI to some extent.

**Figure 2:**
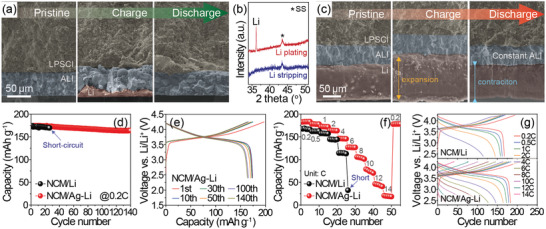
a) Cross‐sectional SEM images of NCM/Ag‐tLi cells showing Li plating/stripping behaviors at the pristine, the first charged, and the first discharged states. b) XRD patterns of deposited/stripped Li on the stainless steel (SS) substrates of the NCM/Ag‐tLi cells shown in (a). For these tests, the SS foil was used as the current collector. c) Cross‐sectional SEM images of the NCM/Ag‐TLi cells showing thickness change in anode during the first cycle. d) Cycling performances of the NCM/Li and NCM/Ag‐Li cells, and e) corresponding voltage profiles of the NCM/Ag‐Li cell at 0.2 C (1 C = 180 mA g^–1^). f) Rate performances and g) corresponding voltage profiles for the NCM/Li and NCM/Ag‐Li cells at different C‐rates from 0.2 to 14 C.

The electrochemical behavior of Li on the ALI was characterized by plating/stripping Li on the ALI shown in Figure 1f. For this study, we chose Li_6_PS_5_Cl (LPSCl) as the SE because argyrodite‐based sulfide SEs have competent ionic conductivity (10^−2^–10^−3^ S cm^−1^) at room temperature and show good mechanical properties.^[^ ^34^, ^35^, ^36^ ^]^ After the charging process (Figure S2a, Supporting Information, for voltage profile), interestingly, a noticeable change in the thickness of the ALI was not detected, and some matter was found beneath the ALI (**Figure** **2**a), which was confirmed as Li by XRD analysis (Figure 2b). Once the ALI is formed, due to the lower adsorption energy of Li on ALI than on Li, the ALI can effectively minimize the energy barrier and thus can facilitate the homogeneous Li deposition during Li plating.^[^ ^29^ ^]^ After that, since the Li tends to be preferentially precipitated on the current collector showing high electrical conductivity than on the insulating LPSCl,^[^ ^37^, ^38^ ^]^ it seems to be detected as metallic Li between the ALI and SS substrate. In the same context, it is strongly believed that the cell with Ag‐TLi would also exhibit similar Li deposition behavior between the ALI and Li, as will be shown in Figure 2c. In addition, reversible Li behavior was observed via clear disappearance of the deposited Li after discharging (Figure 2a). This finding implies that Li is not plated on top of the ALI, but is mostly deposited/stripped at the bottom region, thus being capable of alleviating the short‐circuit and facilitating reversible Li operation of the cell. The stable cycling of the NCM/Ag‐tLi cell during 25 cycles supports this behavior of Li (Figure S3, Supporting Information). Moreover, even in the cells consisting of the Ag‐Li alloy anode with thick residual Li (Figure S2b, Supporting Information, for voltage profile), reversible expansion/contraction of the Li part below the ALI during charging and discharging processes were shown without a thickness change in the ALI (Figure 2c and Figure S4, Supporting Information, for EDS elemental mapping). This validates that the ALI can play the critical role as an intermediate layer, preventing internal short‐circuit and facilitating uniform Li deposition, which will be discussed in more detail later. Additionally, unless otherwise noted, hereafter, we used 150 µm thick Li as a Li source to make the Ag‐Li alloy (denoted as “Ag‐Li”) and supply sufficient Li for better electrochemical performance for further study.

**Figure 3:**
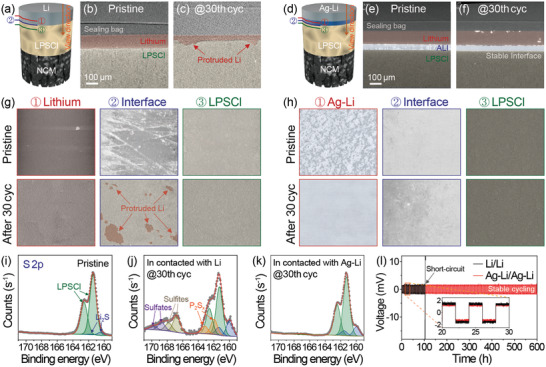
a,d) Schematic illustrations showing view direction and detection points. XRM images of the NCM/Li and NCM/Ag‐Li cells at the pristine state and after 30 cycles: b,c,e‐f) Cross‐sectional view and g,h) top‐view images at the designated points shown in (a) and (d). S 2p XPS spectra of the i) pristine LPSCl, j) LPSCl in contact with Li, and k) LPSCl in contact with Ag‐Li after 30 cycles. l) Voltage profiles of Li/Li and Ag‐Li/Ag‐Li symmetric cells at 0.5 mA cm^–2^ with a fixed capacity of 1 mAh cm^–2^.

**Figure 4:**
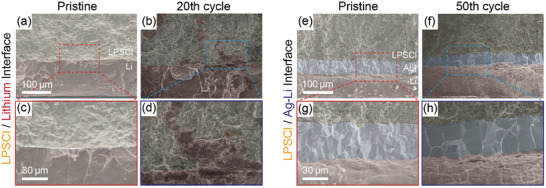
Cross‐sectional low (top row) and high (bottom row) magnified SEM images of the a–d) LPSCl/Li and e–h) LPSCl/Ag‐Li interfaces of the NCM cells with Li metal and Ag‐Li alloy anode at the pristine state and after 20 and 50 cycles.

The effect of the Ag‐Li alloy containing the ALI manifests as an improvement in the cycling performance (Figure 2d). At 0.2 C, when paired with a high‐Ni layered LiNi_0.6_Co_0.2_Mn_0.2_O_2_ (NCM) cathode, the Li cell showed a short‐circuit phenomenon only at the 27th cycle (Figure S5, Supporting Information, voltage profiles), whereas the Ag‐Li cell exhibited a stable cycle life for over 140 cycles without any short‐circuit problem (Figure 2e for voltage profile). The merit of the Ag‐Li alloy anode was also highlighted in the rate performance (Figure 2f). Upon the application of different C‐rates (0.2–14 C), the Ag‐Li cell maintained higher capacities than its counterpart (Figure 2g). In particular, even under a 30‐fold increase in current density, namely, at 6 C, the Ag‐Li cell showed 127 mAh g^–1^ corresponding to 69.5% capacity retention with respect to the result at 0.2 C, whereas the Li cell showed a short‐circuit only at the early stage of 6 C due to the nonuniform Li deposition as shown in the next X‐ray microscopy (XRM) and SEM data (**Figures** **3** and **4**). This remarkable rate performance of the Li‐Ag cell is presumably ascribed to better ionic transport from the stable SE/ALI interface originating from decent interfacial stability at the SE/ALI interface suppressing the formation of insulating side products compared to that of the Li cell case, as will be discussed in detail in Figure 3. These series of electrochemical results confirm the interfacial stability of the Ag‐Li alloy anode in the ASSBs at high current densities. Besides, a smaller resistance of the Ag‐Li cell compared to the Li cell at the 25th cycle in Figure 2f (Figure S6, Supporting Information, for electrochemical impedance spectroscopy (EIS) spectra), the robust cycle stabilities for over 200 cycles without short‐circuit even at a high rate of 12 C (Figure S7, Supporting Information), and decent cycling performance of a high loading cell showing over 70 cycles (Figure S8, Supporting Information) also support this point.

To elucidate the reason for the improved cycling performance of the Ag‐Li cell through the direct evidences, we first conducted the morphological analysis in the liquid cell environment. Under the test condition without external pressure (Figure S9, Supporting Information), the Li cell showed dendritic and uneven Li deposition behavior, whereas uniformly deposited Li was observed in the aged Ag‐Li alloy cell. A similar feature was also reflected in the ASSBs. To characterize the internal morphological variations at the SE/anode contact interface of the cell, XRM analysis was performed to detect the physical changes inside the cell without any damage (Figure 3a–h).^[^ ^11^, ^15^, ^39^ ^]^ According to the cross‐sectional XRM images (Figure 3b,c,e,f), before cycling, both cells exhibited a uniform and flat interface, and the ALI and Li layer were clearly distinguished from each other by noticeable contrast differences in the Ag‐Li cell. However, evident distinctions were observed between these two cells after 30 cycles. Although Li filaments were not detected due to resolution limitations, the Li cell showed a non‐uniform SE/Li interface as shown in Figure 3c, displaying protruded Li inside the SE owing to uneven Li plating or crack generation that provides additional room for Li storage during cycling.^[^ ^12^, ^13^, ^14^, ^15^, ^16^ ^]^ In contrast, the Ag‐Li cell exhibited a uniform SE/Ag‐Li interface even after 30 cycles. These features were more conspicuous in the top‐viewed XRM image obtained along the vertical direction (Figure 3a,d). In the case of the Li cell, the uneven and protruded Li were distinguished at the SE/Li interface, which was well consistent with previous studies reporting short‐circuits caused by dendritic Li.^[^ ^12^, ^15^, ^39^, ^40^ ^]^ Conversely, the Ag‐Li cell maintained a uniform SE/ALI interface analogous to the pristine one after repeated charge and discharge, again potentially because of the lithophilic character of the ALI guiding uniform Li deposition.^[^ ^29^ ^]^ The differences between these two anodes also noted in the continuous long‐lasting Li plating test at 0.2 mA cm^–2^ (Figure S10, Supporting Information). While the internal short‐circuit was identified only near 10 h in the Li/Li symmetric cell, the Ag‐Li/Ag‐Li cell exhibited stable Li plating without short‐circuit even until the active Li of the Ag‐Li counter electrode was almost consumed. A series of these results revealed that the lithiophilic ALI is responsible for the stable cycling of the cell by inducing uniform Li plating and stripping. In addition, Figure S10 (Supporting Information) shows that ≈36 µm Li out of the pristine 150 µm Li was consumed to form the ALI, which was reasonable considering the thickness of the formed ALI (≈45 µm),^[^ ^24^, ^41^ ^]^ consequently implying that ≈114 µm active Li can participate in the electrochemical reactions.

The interfacial stability between ALI and SE can be also linked to the long‐term cycle stability of the Ag‐Li cell. To analyze the chemical composition of the SE surface attached to the anode, S 2p XPS analysis was conducted at the pristine state and after 30 cycles (Figure 3i–k). Even though both cells generated a small amount of side products merely when in contact with SE due to thermodynamic instability (Figure S11, Supporting Information),^[^ ^4^, ^42^, ^43^ ^]^ the Li cell displayed newly formed peaks related to insulating decomposition products such as Li_2_S, Li_2_S*_n_*, and P_2_S*_x_* after 30 cycles, resulting from the interfacial unwanted reactions.^[^ ^17^, ^35^, ^43^, ^44^, ^45^ ^]^ This is in sharp contrast with the 30 cycled Ag‐Li cell exhibiting almost identical peaks to the pristine one. The P 2p XPS result of the Ag‐Li cell in Figure S12 (Supporting Information) also displays no side products including phosphate and P_2_S*_x_*,^[^ ^46^, ^47^ ^]^ showing similar trends to those of the S 2p spectra. Furthermore, the small increase in impedance compared to that of the control case during cycling further supports the interfacial stability of the Ag‐Li cell (Figure S13, Supporting Information). Therefore, we strongly believe that the decent interfacial stability of the ALI to the sulfide‐based SE reduced chemical strain by mitigating parasitic reactions at the SE interface,^[^ ^48^ ^]^ thereby alleviating the crack generation and preventing the short‐circuit caused by Li filament growth. Accordingly, owing to these impressive features, including being lithiophilic, being dendrite‐free, and showing improved interfacial stability, the Ag‐Li symmetric cell achieved a lower overpotential and better cycling performance up to 150 cycles compared to the control cell (Figure 3l). In the same manner, these consistent microscopic and spectroscopic results, showing the uniformity and stability at the SE/Ag‐Li interface, demonstrate the aforementioned improved electrochemical performances of Ag‐Li cells shown in Figure 2, as well.

To reconfirm the stable integrity of the ALI/SE interface in the Ag‐Li cell after prolonged cycles, we finally visualized the cross‐sectional SEM images (**Figure** 4). At the pristine state, both cells displayed a flat and uniform SE/anode interface as identified in the XRM data. However, the Li cell exhibited the protruded matters strongly believed to be Li in the SE, as previously reported, after only 20 cycles,^[^ ^12^, ^15^, ^49^ ^]^ whereas the Ag‐Li cell showed well‐preserved interfacial morphology similar to that of the pristine state without any change in the ALI thickness after 50 cycles. Furthermore, even after 80 cycles, for the Ag‐Li cell, the interface was stably maintained without any Li filaments or protruded Li inside the SE (Figure S14, Supporting Information). These supportive analytical results verify once again that, owing to the lithiophilic nature of the in situ formed ALI, the ALI physically can guide the uniform Li deposition beneath the ALI, and chemically mitigate the interfacial side reactions between ALI and SE, thus contributing to the reversible Li operation and long‐term cycling of ASSBs.

## 3 Conclusion

In summary, we investigated the interfacial stabilizing effect of in situ formed ALI that was made using a mass‐producible roll pressing method on the electrochemical performance of ASSBs. The physical and chemical changes at the SE/anode interface were characterized using various techniques, such as XRM, XPS, and SEM. After cycling, the cell with the Li metal showed protruded Li inside the SE and decomposed SE at the SE/Li interface. Conversely, the cell incorporating the Ag‐Li alloy anode exhibited a uniform and stable SE/Ag‐Li interface, owing to the in situ formed intermetallic layer and residual lithiophilic Ag component, enabling the maintenance of a stable interface and the uniform Li deposition. Because of these advantages, the ASSB containing the ALI not only showed superior cyclability compared to the Li cell exhibiting frequent initial short‐circuit, but also showed a stable cycle life even at a high rate of 12 C. This work delivers an invaluable lesson that, based on an easy and highly productive process, an anode surface engineering through the introduction of a functional layer that can be in situ formed for a dendrite‐free and stable interface without further processes can be a viable option for realizing the practical application of ASSBs.

## 4 Experimental Section

All experimental details are included in the Supporting Information.

## Conflict of Interest

The authors declare no conflict of interest.

## Supporting information

Supporting Information

## Data Availability

Research data are not shared.

## References

[1] J. Janek , W. G. Zeier , Nat. Energy 2016, 1, 16141.

[2] N. Kamaya , K. Homma , Y. Yamakawa , M. Hirayama , R. Kanno , M. Yonemura , T. Kamiyama , Y. Kato , S. Hama , K. Kawamoto , A. Mitsui , Nat. Mater. 2011, 10, 682. 21804556 10.1038/nmat3066

[3] A. Manthiram , X. Yu , S. Wang , Nat. Rev. Mater. 2017, 2, 16103.

[4] A. Banerjee , X. Wang , C. Fang , E. A. Wu , Y. S. Meng , Chem. Rev. 2020, 120, 6878. 32603100 10.1021/acs.chemrev.0c00101

[5] Q. Zhao , S. Stalin , C.‐Z. Zhao , L. A. Archer , Nat. Rev. Mater. 2020, 5, 229.

[6] M. Ali , C.‐H. Doh , Y.‐J. Lee , B.‐G. Kim , J.‐W. Park , J. Park , G. Park , W.‐J. Lee , S.‐M. Lee , Y.‐C. Ha , Energy Technol. 2021, 9, 2001096.

[7] L. Porz , T. Swamy , B. W. Sheldon , D. Rettenwander , T. Frömling , H. L. Thaman , S. Berendts , R. Uecker , W. C. Carter , Y.‐M. Chiang , Adv. Energy Mater. 2017, 7, 1701003.

[8] A. Ferrese , J. Newman , J. Electrochem. Soc. 2014, 161, A1350.

[9] C. Monroe , J. Newman , J. Electrochem. Soc. 2003, 150, A1377.

[10] C. Monroe , J. Newman , J. Electrochem. Soc. 2005, 152, A396.

[11] J.‐M. Doux , H. Nguyen , D. H. S. Tan , A. Banerjee , X. Wang , E. A. Wu , C. Jo , H. Yang , Y. S. Meng , Adv. Energy Mater. 2020, 10, 1903253.

[12] M. Sun , T. Liu , Y. Yuan , M. Ling , N. Xu , Y. Liu , L. Yan , H. Li , C. Liu , Y. Lu , Y. Shi , Y. He , Y. Guo , X. Tao , C. Liang , J. Lu , ACS Energy Lett. 2021, 6, 451.

[13] L. Ye , X. Li , Nature 2021, 593, 218. 33981053 10.1038/s41586-021-03486-3

[14] J. A. Lewis , F. J. Q. Cortes , M. G. Boebinger , J. Tippens , T. S. Marchese , N. Kondekar , X. Liu , M. Chi , M. T. McDowell , ACS Energy Lett. 2019, 4, 591.

[15] S. Hao , S. R. Daemi , T. M. M. Heenan , W. Du , C. Tan , M. Storm , C. Rau , D. J. L. Brett , P. R. Shearing , Nano Energy 2021, 82, 105744.

[16] Z. Ning , D. S. Jolly , G. Li , R. De Meyere , S. D. Pu , Y. Chen , J. Kasemchainan , J. Ihli , C. Gong , B. Liu , D. L. R. Melvin , A. Bonnin , O. Magdysyuk , P. Adamson , G. O. Hartley , C. W. Monroe , T. J. Marrow , P. G. Bruce , Nat. Mater. 2021, 20, 1121. 33888903 10.1038/s41563-021-00967-8

[17] Z. Shen , W. Zhang , G. Zhu , Y. Huang , Q. Feng , Y. Lu , Small Methods 2020, 4, 1900592.

[18] H.‐D. Lim , J.‐H. Park , H.‐J. Shin , J. Jeong , J. T. Kim , K.‐W. Nam , H.‐G. Jung , K. Y. Chung , Energy Storage Mater. 2020, 25, 224.

[19] B. Wu , S. Wang , W. J. Evans Iv , D. Z. Deng , J. Yang , J. Xiao , J. Mater. Chem. A 2016, 4, 15266.

[20] A. L. Santhosha , L. Medenbach , J. R. Buchheim , P. Adelhelm , Batteries Supercaps 2019, 2, 524.

[21] S. W. Park , G. Oh , J.‐W. Park , Y.‐C. Ha , S.‐M. Lee , S. Y. Yoon , B. G. Kim , Small 2019, 15, 1900235. 10.1002/smll.201900235 30963717

[22] K. Yan , Z. Lu , H.‐W. Lee , F. Xiong , P.‐C. Hsu , Y. Li , J. Zhao , S. Chu , Y. Cui , Nat. Energy 2016, 1, 16010.

[23] C. Jin , O. Sheng , J. Luo , H. Yuan , C. Fang , W. Zhang , H. Huang , Y. Gan , Y. Xia , C. Liang , J. Zhang , X. Tao , Nano Energy 2017, 37, 177.

[24] B. G. Kim , D. W. Kang , G. Park , S. H. Park , S.‐M. Lee , J. W. Choi , Chem. Eng. J. 2021, 422, 130017.

[25] T. Liu , Q. Hu , X. Li , L. Tan , G. Yan , Z. Wang , H. Guo , Y. Liu , Y. Wu , J. Wang , J. Mater. Chem. A 2019, 7, 20911.

[26] S. Jin , Y. Ye , Y. Niu , Y. Xu , H. Jin , J. Wang , Z. Sun , A. Cao , X. Wu , Y. Luo , H. Ji , L.‐J. Wan , J. Am. Chem. Soc. 2020, 142, 8818. 32310653 10.1021/jacs.0c01811

[27] K. Lu , H. Xu , H. He , S. Gao , X. Li , C. Zheng , T. Xu , Y. Cheng , J. Mater. Chem. A 2020, 8, 10363.

[28] Y.‐G. Lee , S. Fujiki , C. Jung , N. Suzuki , N. Yashiro , R. Omoda , D.‐S. Ko , T. Shiratsuchi , T. Sugimoto , S. Ryu , J. H. Ku , T. Watanabe , Y. Park , Y. Aihara , D. Im , I. T. Han , Nat. Energy 2020, 5, 299.

[29] L.‐N. Wu , J. Peng , F.‐M. Han , Y.‐K. Sun , T. Sheng , Y.‐Y. Li , Y. Zhou , L. Huang , J.‐T. Li , S.‐G. Sun , J. Mater. Chem. A 2020, 8, 4300.

[30] A. Dębski , S. Terlicka , A. Budziak , W. Gąsior , J. Alloys Compd. 2018, 732, 210.

[31] M. H. Braga , A. Dębski , S. Terlicka , W. Gąsior , A. Góral , J. Alloys Compd. 2020, 817, 152811.

[32] V. V. Pavlyuk , G. S. Dmytriv , I. I. Tarasiuk , I. V. Chumak , H. Pauly , H. Ehrenberg , Solid State Sci. 2010, 12, 274.

[33] S. Terlicka , A. Dębski , A. Budziak , M. Zabrocki , W. Gąsior , Thermochim. Acta 2019, 673, 185.

[34] S. Boulineau , M. Courty , J.‐M. Tarascon , V. Viallet , Solid State Ionics 2012, 221, 1.

[35] X. Bai , Y. Duan , W. Zhuang , R. Yang , J. Wang , J. Mater. Chem. A 2020, 8, 25663.

[36] M.‐J. Kim , J.‐W. Park , B. G. Kim , Y.‐J. Lee , Y.‐C. Ha , S.‐M. Lee , K.‐J. Baeg , Sci. Rep. 2020, 10, 11923. 32681025 10.1038/s41598-020-68885-4 PMC7367834

[37] K. H. Park , D. W. Kang , J.‐W. Park , J.‐H. Choi , S.‐J. Hong , S. H. Song , S.‐M. Lee , J. Moon , B. G. Kim , J. Mater. Chem. A 2021, 9, 1822.

[38] J. Li , P. Zou , S. W. Chiang , W. Yao , Y. Wang , P. Liu , C. Liang , F. Kang , C. Yang , Energy Storage Mater. 2020, 24, 700.

[39] J. Tippens , J. C. Miers , A. Afshar , J. A. Lewis , F. J. Q. Cortes , H. Qiao , T. S. Marchese , C. V. Di Leo , C. Saldana , M. T. McDowell , ACS Energy Lett. 2019, 4, 1475.

[40] H. Park , J. Kim , D. Lee , J. Park , S. Jo , J. Kim , T. Song , U. Paik , Adv. Sci. 2021, 8, 2004204. 10.1002/advs.202004204 PMC8188223 34105278

[41] J. A. Rodriguez , J. Hrbek , J. Phys. Chem. 1994, 98, 4061.

[42] W. D. Richards , L. J. Miara , Y. Wang , J. C. Kim , G. Ceder , Chem. Mater. 2016, 28, 266.

[43] S. Wenzel , S. J. Sedlmaier , C. Dietrich , W. G. Zeier , J. Janek , Solid State Ionics 2018, 318, 102.

[44] A. D. Bui , S.‐H. Choi , H. Choi , Y.‐J. Lee , C.‐H. Doh , J.‐W. Park , B. G. Kim , W.‐J. Lee , S.‐M. Lee , Y.‐C. Ha , ACS Appl. Energy Mater. 2021, 4, 1.

[45] D. H. S. Tan , E. A. Wu , H. Nguyen , Z. Chen , M. A. T. Marple , J.‐M. Doux , X. Wang , H. Yang , A. Banerjee , Y. S. Meng , ACS Energy Lett. 2019, 4, 2418.

[46] J. Auvergniot , A. Cassel , J.‐B. Ledeuil , V. Viallet , V. Seznec , R. Dedryvère , Chem. Mater. 2017, 29, 3883.

[47] J. Auvergniot , A. Cassel , D. Foix , V. Viallet , V. Seznec , R. Dedryvère , Solid State Ionics 2017, 300, 78.

[48] H.‐K. Tian , A. Chakraborty , A. A. Talin , P. Eisenlohr , Y. Qi , J. Electrochem. Soc. 2020, 167, 090541.

[49] J. Kasemchainan , S. Zekoll , D. S. Jolly , Z. Ning , G. O. Hartley , J. Marrow , P. G. Bruce , Nat. Mater. 2019, 18, 1105. 31358941 10.1038/s41563-019-0438-9

