# Supplementary material for: In Situ Formed Ag‐Li Intermetallic Layer for Stable Cycling of All‐Solid‐State Lithium Batteries
Source: Adv Sci (Weinh). 2021 Nov 21;9(1):2103826. doi: 10.1002/advs.202103826 (PMC8728838; doi:10.1002/advs.202103826)
Supplement: Supplementary file 1 — Supporting Information [file ADVS-9-2103826-s001.pdf]

WILEY-VCH

© 2021 Wiley-VCH GmbH

ADVANCED SCIENCE
Open Access

Supporting Information

for *Adv. Sci.*, DOI: 10.1002/advs.202103826

In situ Formed Ag-Li Intermetallic Layer for Stable Cycling of All-solid-state Lithium Batteries

Hong Jun Choi, Dong Woo Kang, Jun-Woo Park, Jun-Ho Park, Yoo-Jun Lee, Yoon-Cheol Ha, Sang-Min Lee, Seog Young Yoon, and Byung Gon Kim**

Supporting Information

In situ Formed Ag-Li Intermetallic Layer for Stable Cycling of All-solid-state Lithium Batteries

Hong Jun Choi, Dong Woo Kang, Jun-Woo Park, Jun-Ho Park, Yoo-Jun Lee, Yoon-Cheol Ha, Sang-Min Lee, Seog Young Yoon, and Byung Gon Kim**

H. J. Choi, D. W. Kang, Dr. J.-W. Park, Dr. Y.-J. Lee, Dr. Y.-C. Ha, Dr. S.-M. Lee,[†] Dr. B. G. Kim
Next Generation Battery Research Center, Korea Electrotechnology Research Institute (KERI), 12, Jeongiui-gil, Seongsan-gu, Changwon-si, Gyeongsangnam-do 51543, Republic of Korea
E-mail: byunggonkim@keri.re.kr

H. J. Choi, Prof. S. Y. Yoon
School of Materials Science and Engineering, Pusan National University, 2, Busandaehak-ro 63beon-gil, Geumjeong-gu, Busan, 46241, Republic of Korea
E-mail: syy3@pusan.ac.kr

[†]Present address: Graduate Institute of Ferrous & Energy Materials Technology, Pohang University of Science and Technology (POSTECH), 77 Cheongam-Ro, Nam-gu, Pohang, Gyeongbuk 37673, Republic of Korea

Experimental Section

Materials: Ag foil (thickness = 10 μm, 99.98%) was purchased from Nilaco (Japan). Li metal foil (thickness = 40, 150, and 250 μm) was purchased from Honjo (Japan). Stainless steel foil (SS, thickness = 10 μm) was purchased from MTI Korea (Korea). Li_2S (99.98%), P_2S_5 (99%), and LiCl (99%) were purchased from Sigma-Aldrich (USA). $LiNi_{0.6}Co_{0.2}Mn_{0.2}O_2$ (NCM, particle diameter: 4 μm) was provided by the L&F Co. (Korea). Graphitic hollow nanocarbon (GHC) was obtained using heat treatment of Super P (TIMCAL, Switzerland) at 2400 ^{o}C.[S1] In this study, the GHC was used as a conducting agent because it can mitigate the interfacial side reactions between the solid electrolyte (SE) and additive.

Preparation of Ag-Li Alloy and LPSCl Solid Electrolyte: The Ag-Li alloy foil was prepared by roll pressing using Ag and Li foils, and the as-prepared foil was kept under external pressure to complete the Ag-Li alloying reaction using a jig for 2 days. The sulfide SE, argyrodite Li_6PS_5Cl (LPSCl), was prepared based on the following procedure:[S2,S3] A stoichiometric mixture of Li_2S, P_2S_5, and LiCl was mechanically ball-milled at 600 rpm for 10 h using a high energy ball-milling machine (Pulverizett7, Fritsch, USA). After finishing the milling process, the vessel was transferred into a glove box, and the LPSCl powder was obtained.

Test Cell Assembly and Electrochemical Tests: Prior to preparing the composite cathode, all materials were dried under vacuum at 80 °C overnight, and then, transferred into an Ar-filled glovebox. To obtain a homogeneous mixture, the composite cathode was prepared by hand-mixing the NCM cathode material, LPSCl solid electrolyte, and GHC conducting agent in a weight ratio of 75:23:2, using an agate mortar for 40 min. To test the electrochemical performance, the homemade pressurized cell was assembled.[S1,S4] First, 200 mg of the SE was placed in the polyether ether ketone mold (diameter: 14 mm) and cold pressed with 0.5 ton for 1 min. Then, 26.7 or 49.3 mg (loading: 13 or 24 mg_{NCM} cm^{-2}, areal capacity: 2.3 or 4.3 mAh cm^{-2}) of cathode composite was filled on the SE pellet and cold pressed with 4 tons for 3 min.

The Ag-Li alloyed foil with an SS current collector was attached to the other side of the SE pellet. For the Li deposition test below the in situ formed Ag-Li intermetallic layer (ALI), 26.7 mg of cathode composite (areal capacity = 2.3 mAh cm^{-2}) was used to supply active Li, thus detecting a noticeable change in the anode thickness on the SS foil. All fabrication steps were conducted in an Ar-filled glove box and dry room at a dew point of approximately –70 °C. The cell tests were carried out using a battery cycler (WBCS3000L, WonAtech, Korea) at 55 °C. For the cycling performance test, the galvanostatic charge/discharge modes were used at current densities of 0.2 and 0.5 C in the voltage range of 2.4 – 4.25 V vs. Li/Li^+. For the rate capability test, the unit cells were cycled at a charging rate of 0.2 C and different discharging rates from 0.2 to 14 C (1 C = 180 mA g^{-1}), and thereafter, cycled at 0.2 C. In order to access the symmetric cell, the Ag-Li alloy or Li foil was attached to both sides of the SE pellet. Electrochemical impedance spectroscopy (EIS) analysis was performed in the frequency range of 10 mHz to 7 MHz at 25 °C using a VSP tester (Biologic, France).[S1,S5-S7]

Characterization: To characterize the Ag-Li alloyed phase and morphological changes of the electrode during electrochemical tests, the cycled cells were disassembled in a glovebox; then, the pelleted electrodes were analyzed using an X-ray diffractometer (X'pert Pro, PANalytical, Netherlands) with Cu K_α (λ = 0.15406 nm) and field-emission scanning electron microscopy (FE-SEM, S-4800, Hitachi, Japan). For the X-ray diffraction (XRD) analysis, a gastight XRD holder (PANalytical, Netherlands) was used to avoid air exposure. The elemental mapping of the electrodes was performed using energy-dispersive X-ray spectroscopy (EDS, Sirion, FEI, USA). The chemical compositions and bonding characteristics of the LPSCl surface were analyzed using X-ray photoelectron spectroscopy (XPS, K-alpha, Thermo Scientific, England) with an Al K_α line as the X-ray source. The C 1s peak (284.8 eV) was used as reference. For the XPS characterization, we used 250 μm thick Li for cell fabrication to easily detach the anode from the pelleted cell after electrochemical tests. A vacuum transfer module (Thermo Scientific, England) was used to avoid air contamination. X-ray microscopy (XRM,

Xradia 520 Versa, ZEISS, Germany) with a source voltage of 80 kV and a power of 7 W using the Air filter was employed to analyze the internal interfacial morphology of the electrode without cell fracture. A 4× objective was used with an exposure of 3 s and 2401 projections, giving a resolution of about 0.8408 μm.

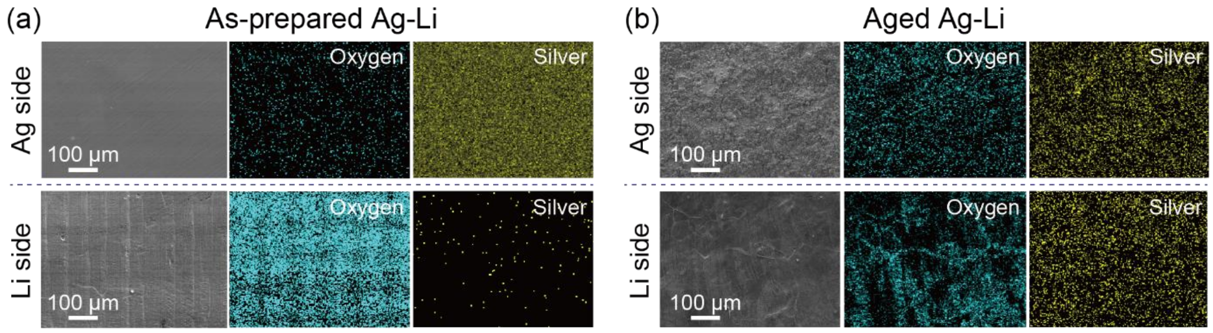

Figure S1. Top-view SEM images of the as-prepared Ag-Li and aged Ag-Li alloys along with EDS elemental mapping images of oxygen and silver.

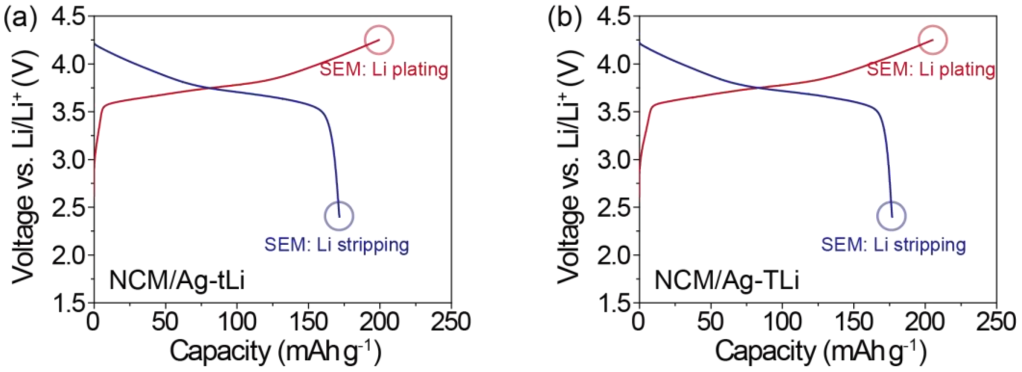

Figure S2. The 1st voltage profiles of the NCM/Ag-tLi and NCM/Ag-TLi cells at 0.2 C for the SEM analysis shown in Figures 2a and c.

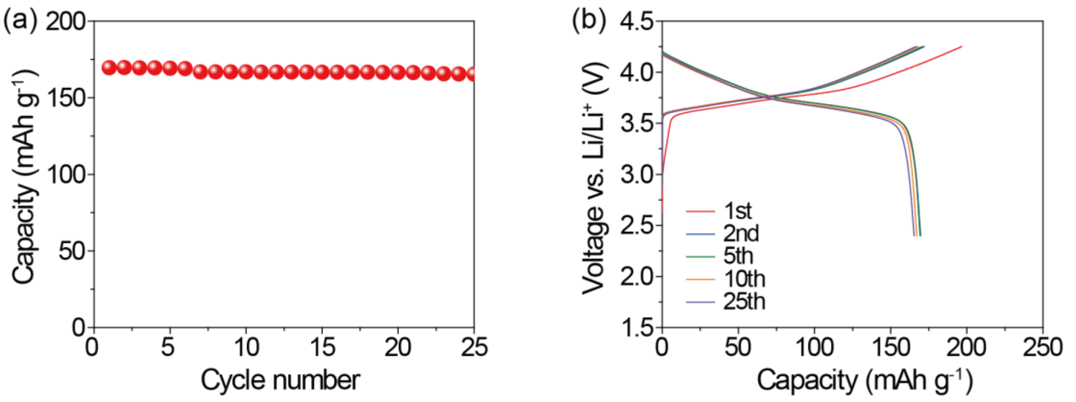

Figure S3. (a) Cycling performance and (b) corresponding voltage profiles of the NCM/Ag-tLi cells at 0.2 C.

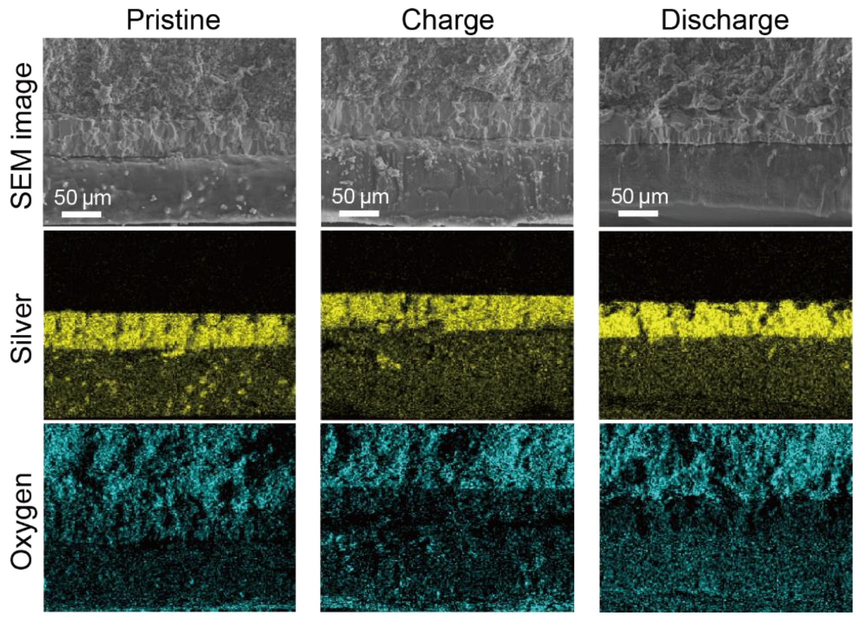

Figure S4. Cross-sectional SEM images of the NCM/Ag-Li cells along with EDS elemental mappings of silver and oxygen during the 1st cycle shown in Figure 2c.

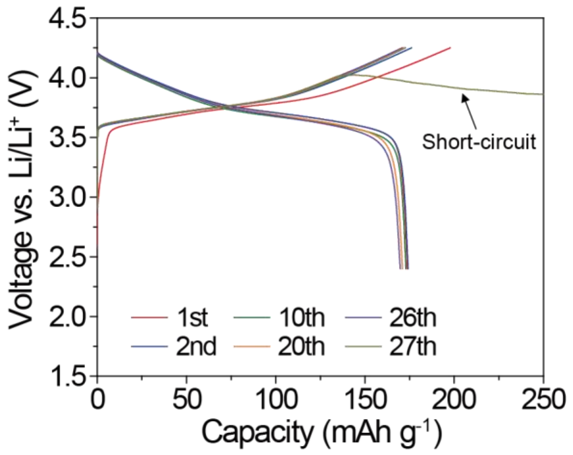

Figure S5. Voltage profiles of the NCM/Li cell shown in Figure 2d.

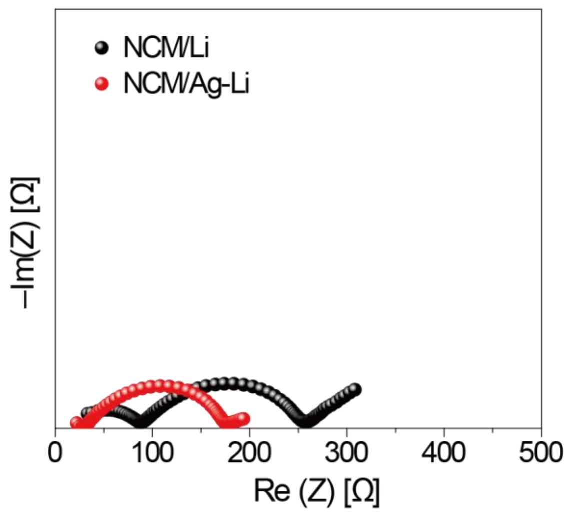

Figure S6. Overlapped EIS spectra of NCM/Li and NCM/Ag-Li cells at the 25th charged state during rate performance test shown in Figure 2f, showing a smaller resistance for the NCM/Ag-Li cell compared to the NCM/Li cell. This confirms the stable SE/Ag-Li interface at high current densities.

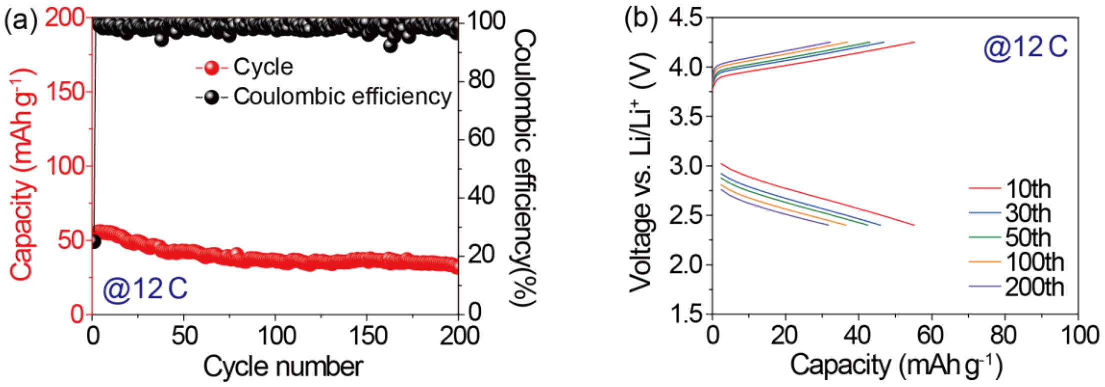

Figure S7. (a) Cycling performance and corresponding coulombic efficiency of the NCM/Ag-Li cell at 12 C, and (b) their corresponding voltage profiles. To generate a stable interface in the cell, the cell was precycled at a charging rate of 0.2 C and a discharging rate of 12 C at the first cycle.

Note: The initial CE is low because the charge capacity at 0.2 C is much higher than that of the discharge capacity at 12 C due to the large discharging overpotential during the precycling process to form a stable interface in the cell. In addition, side reactions that occur during the first charging process can affect the low initial CE.[S3,S8]

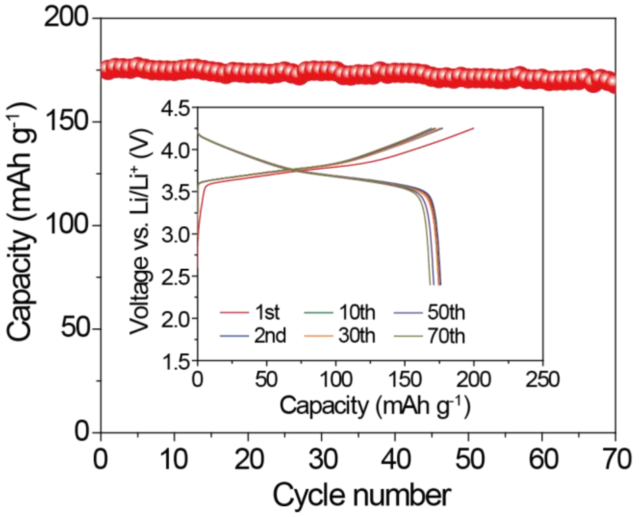

Figure S8. Cycling performance and (inset) corresponding voltage profiles of the NCM/Ag-Li cell with a practical-loading density of 24 mg_{NCM} cm^{-2} at 0.2 C.

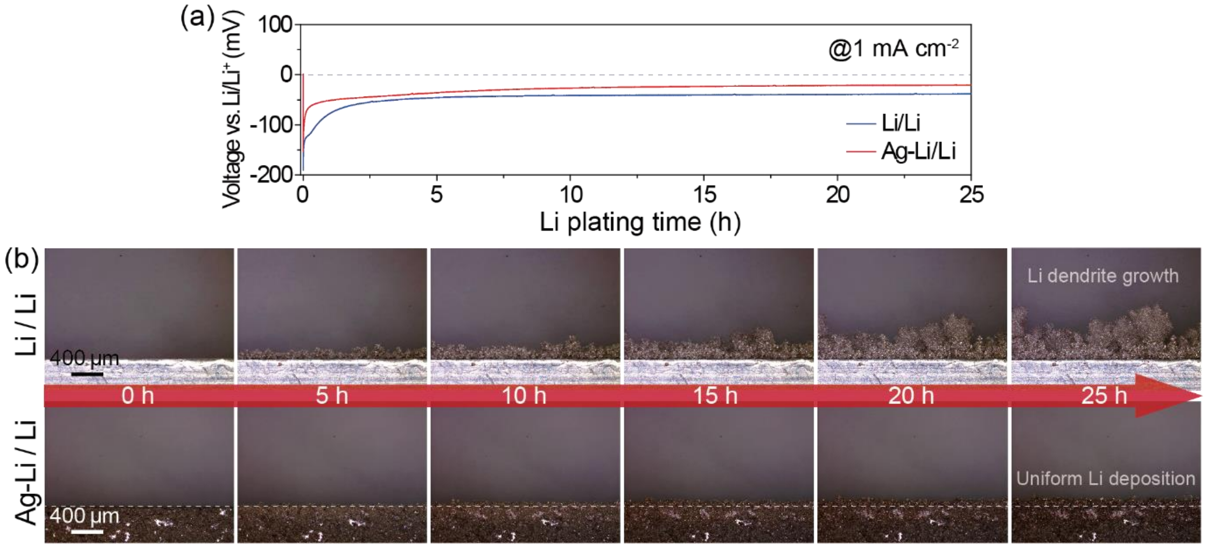

Figure S9. (a) Voltage profile of the Li/Li symmetric and Ag-Li/Li cells during Li plating at 1 mA cm^{-2}, and (b) corresponding optical microscope images of the Li/Li symmetric and Ag-Li /Li cells taken at various times during Li deposition.

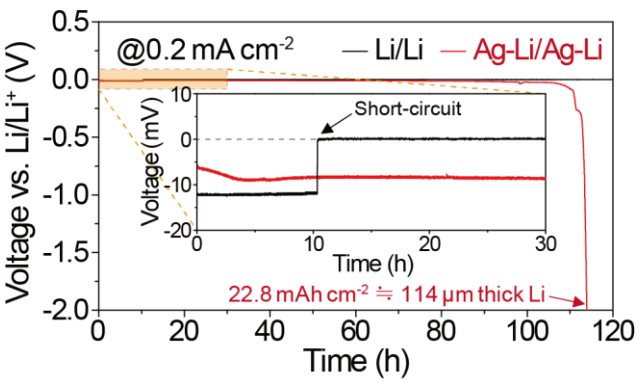

Figure S10. Voltage profiles of the Li/Li and Ag-Li/Ag-Li symmetric cells at 0.2 mA cm^{-2} during long-lasting Li plating. For this test, 150 μm thick Li was used to fabricate the Ag-Li alloy.

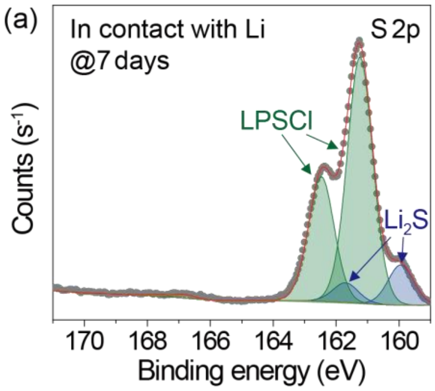

Figure S11. S 2p XPS spectra of the LPSCl in contact with the (a) Li metal and (b) Ag-Li alloy after 7 days.

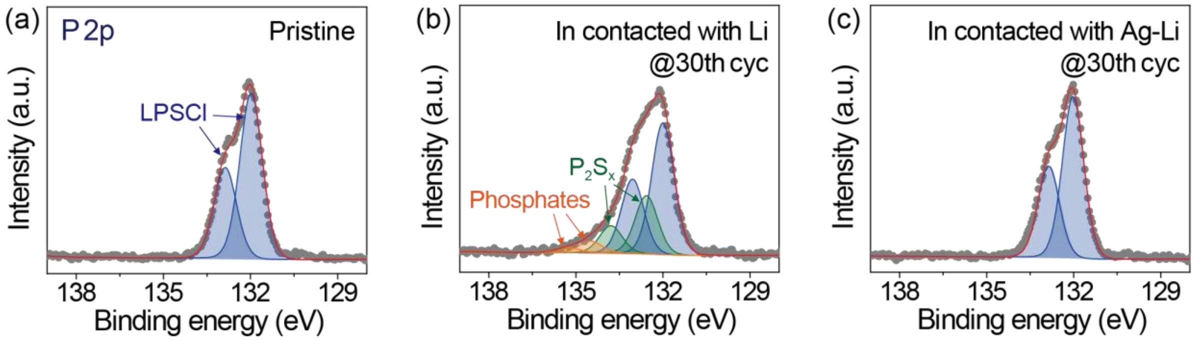

Figure S12. P 2p XPS spectra of the (a) pristine LPSCl, (b) LPSCl in contact with Li, and (c) LPSCl in contact with Ag-Li after 30 cycles. The LPSCl of Li cell exhibits the decomposition products such as P_2S_x and phosphates,[S3,S9] resulting from the interfacial side reactions.

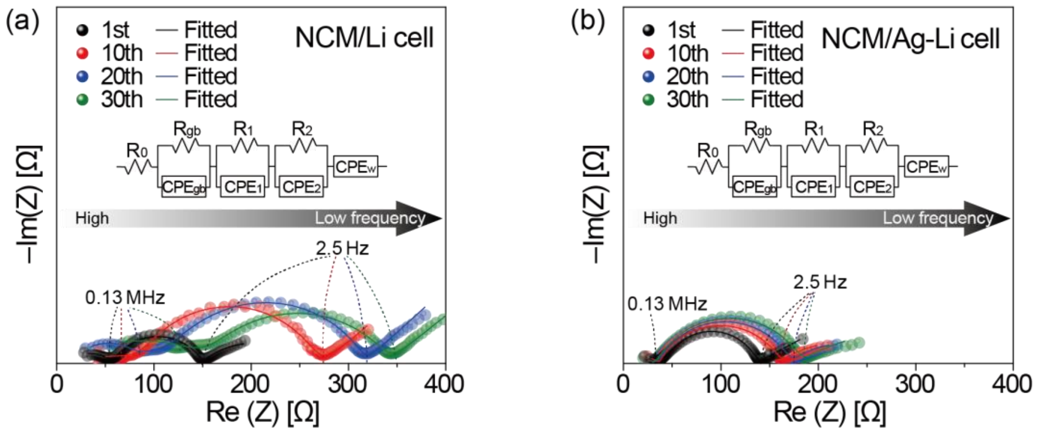

Figure S13. Nyquist plots for the (a) NCM/Li and (b) NCM/Ag-Li cells shown in Figure 2d. The NCM/Ag-Li cell showed smaller resistances than those of NCM/Li cell at the charged state during 30 cycles, supporting the stable interface at the anode side as shown in Figure 3k. The inset indicates an equivalent circuit model.[S10,S11] (R_0: SE resistance. R_{gb}: Grain boundary resistance of the SE. R_1 and R_2: Charge transfer resistance at the cathode and anode interfaces, respectively. CPE_1 and CPE_2: Constant phase elements at the cathode and anode side, respectively. CPE_w: Warburg element, indicating the solid-state diffusion process of Li^+ in the NCM particles)

Note: To further demonstrate the interfacial stability of the ALI to the sulfide-based SE, EIS tests were carried out over 30 cycles. Figure S13 displays the EIS spectra of Li and Ag-Li cells at 25 °C. The combined semicircle in the high- and middle-frequency regions is attributed to the small grain boundary resistance of the SE (R_{gb}) and large cathode/SE interfacial resistance (R_1), respectively. The semicircle in the low-frequency region is correlated with the charge transfer resistance between the anode and SE (R_2). The x-axis intercept is assigned to the resistance of the SE layer (R_0), which corresponds to the bulk resistance of the SE. While the Li cell showed a conspicuous increase in impedance, a small increase in impedance was observed in the Ag-Li cell. Considering the cathode and SE are the same in the Li and Ag-Li cells, in the low-frequency region, the result of the Li cell is mainly due to the increase in the resistance caused by the interfacial side reactions at the anode/SE interface, and the Ag-Li cell exhibited a relatively small increase in resistance because it maintained a stable anode interface during cycling. This spectroscopic result supports the interfacial stability at the anode side in the Ag-Li cell.

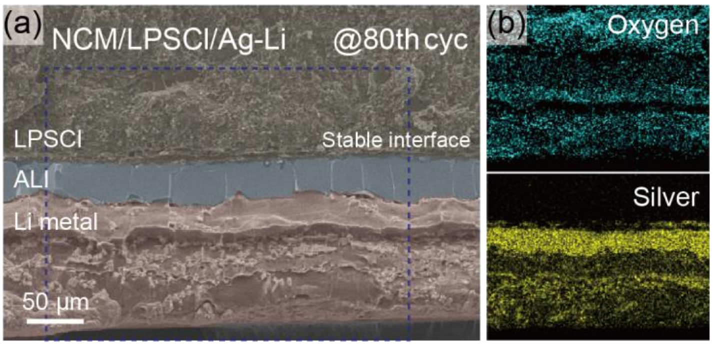

Figure S14. (a) Cross-sectional SEM image of the NCM cell with Ag-Li alloy anode after 80 cycles and (b) corresponding EDS elemental mappings of oxygen and silver from the inset blue box in (a).

References

[S1] S. W. Park, G. Oh, J.-W. Park, Y.-C. Ha, S.-M. Lee, S. Y. Yoon, B. G. Kim, *Small* **2019**, *15*, 1900235.

[S2] S. Boulineau, M. Courty, J.-M. Tarascon, V. Viallet, *Solid State Ion.* **2012**, *221*, 1.

[S3] J. Auvergniot, A. Cassel, J.-B. Ledeuil, V. Viallet, V. Seznec, R. Dedryvère, *Chem. Mater.* **2017**, *29*, 3883.

[S4] A. D. Bui, S.-H. Choi, H. Choi, Y.-J. Lee, C.-H. Doh, J.-W. Park, B. G. Kim, W.-J. Lee, S.-M. Lee, Y.-C. Ha, *ACS Appl. Energy Mater.* **2021**, *4*, 1.

[S5] K. H. Park, D. W. Kang, J.-W. Park, J.-H. Choi, S.-J. Hong, S. H. Song, S.-M. Lee, J. Moon, B. G. Kim, *J. Mater. Chem. A* **2021**, *9*, 1822.

[S6] D. W. Kang, J. Moon, H.-Y. Choi, H.-C. Shin, B. G. Kim, *J. Power Sources* **2021**, *490*, 229504.

[S7] B. G. Kim, C. Jo, J. Shin, Y. Mun, J. Lee, J. W. Choi, *ACS Nano* **2017**, *11*, 1736.

[S8] D. H. S. Tan, E. A. Wu, H. Nguyen, Z. Chen, M. A. T. Marple, J.-M. Doux, X. Wang, H. Yang, A. Banerjee, Y. S. Meng, *ACS Energy Lett.* **2019**, *4*, 2418.

[S9] J. Auvergniot, A. Cassel, D. Foix, V. Viallet, V. Seznec, R. Dedryvère, *Solid State Ion.* **2017**, *300*, 78.

[S10] W. Zhang, D. A. Weber, H. Weigand, T. Arlt, I. Manke, D. Schröder, R. Koerver, T. Leichtweiss, P. Hartmann, W. G. Zeier, J. Janek, *ACS Appl. Mater. Interf.* **2017**, *9*, 17835.

[S11] R. Koerver, I. Aygün, T. Leichtweiß, C. Dietrich, W. Zhang, J. O. Binder, P. Hartmann, W. G. Zeier, J. Janek, *Chem. Mater.* **2017**, *29*, 5574.
